# Mirror Instabilities in the Inner Magnetosphere and Their Potential for Localized ULF Wave Generation

**DOI:** 10.1029/2020JA028773

**Published:** 2021-02-10

**Authors:** M. B. Cooper, A. J. Gerrard, L. J. Lanzerotti, A. R. Soto‐Chavez, H. Kim, I. V. Kuzichev, L. V. Goodwin

**Affiliations:** ^1^ Center for Solar‐Terrestrial Research New Jersey Institute of Technology Newark NJ USA; ^2^ Syntek Technologies Inc. Fairfax VA USA; ^3^ Space Research Institute of Russian Academy of Sciences Moscow Russia; ^4^ Cooperative Programs for the Advancement of Earth System Science University Corporation for Atmospheric Research Boulder CO USA

**Keywords:** inner magnetosphere, magnetotail injections, mirror mode unstable plasma, ULF waves

## Abstract

Results from the NASA Van Allen Probes mission indicate extensive observations of mirror/drift‐mirror (M/D‐M hereafter) unstable plasma regions in the night‐side inner magnetosphere. Said plasmas lie on the threshold between the kinetic and frozen‐in plasma regimes and have favorable conditions for the formation of M/D‐M modes and subsequent ultralow frequency (ULF) wave signatures in the surrounding plasma. We present the results of a climatological analysis of plasma‐*γ* (anisotropy measure) and total plasma‐*β* (ratio of particle to magnetic field pressure) in regard to the satisfaction of instability conditions on said M/D‐M modes under bi‐Maxwellian distribution assumption, and ascertain the most likely region for such plasmas to occur. Our results indicate a strong preference for the premidnight sector of the night‐side magnetosphere, with events ranging in time scales from half a minute (roughly 200 km in scale size) to several hours (multiple Earth radii). The statistical distribution of these plasma regions explicitly identifies the source region of “storm time Pc5 ULF waves” and suggests an alternative mechanism for their generation in the night‐side inner magnetosphere.

## Key Points

- Mirror mode unstable plasmas occur in the dusk‐to‐midnight inner magnetosphere
- Mirror‐unstable plasmas are likely due to magnetotail injections
- Mirror mode propagation can lead to ultra‐low frequency (ULF) mode generation

## 1 Introduction

Several plasma instabilities, including mirror and firehose instabilities (Achterberg, 2013; Gary et al., 1976; Migliuolo, 1983; Schekochihin et al., 2008), populate the regime between frozen‐in and fully kinetic plasma. Understanding such instabilities is vital to understanding fundamental particle‐field energy dynamics of said regime. As has been shown (Bale et al., 2002; Curtis et al., 1982; Tang et al., 2013; Thorne, 2010), plasma instabilities represent a unique degree of freedom for energy transfer in plasmas, and mirror modes are no exception. Hydrogen plasmas whose *β* (particle pressure/magnetic field pressure) values are above unity (high‐*β*) represent plasmas where mirror modes are likely to form. High‐*β* plasmas have been found in the solar wind (Crooker et al., 2003; Ebert et al., 2014), as well as the Earth’s magnetosheath (Tsurutani et al., 1982) and magnetotail/plasma sheet (Boakes et al., 2014; Cao et al., 2006; Walsh et al., 2007).

A presence in the night‐side inner magnetosphere was not established until 2017, when a survey of high‐*β* plasmas within Earth's inner magnetosphere (i.e., *L* < 6) was reported (Cohen et al., 2017) for one full magnetic local time (MLT) precession around Earth's inner magnetosphere using Van Allen Probes spacecraft data. This study found an asymmetry of high‐*β* occurrences, with preference for the dusk‐to‐midnight sector of the inner magnetosphere. It was suggested that inner magnetosphere‐bound plasma injections, a prevalent occurrence in the region, are the source of particle densities and free energy to drive mirror/drift‐mirror mode (M/D‐M) formation. Furthermore, it was speculated that high‐*β* plasmas are a likely source of ULF waves in the dusk‐to‐midnight sector, often associated with “stormtime ULF waves” (Ukhorskiy et al., 2009). The Cohen et al. (2017) study did not consider the physical source of such ULF waves. As such, in this paper we turn our attention to the M/D‐M instabilities (Balikhin et al., 2010; Hasegawa, 1969; Soto‐Chavez et al., 2019; Vedenov & Sagdeev, 1960), which arise in a high‐*β* plasma environment with velocity space anisotropy (*γ* > 0). We assume injections are the source of particle fluxes and anisotropy providing the necessary free energy (*γ* > 0) and plasma environment (*β* > 1) for the formation of mirror modes in the dusk‐to‐midnight sector of the inner magnetosphere.

One of the first papers to explore mirror modes theoretically was in 1960 (Vedenov & Sagdeev, 1960), and investigated the case of a plasma where the oscillation was parallel to the magnetic field line. This theoretical investigation found an instability which resulted in the overall transfer of the kinetic free energy of the particles from transverse motion to longitudinal motion, until the two motional states reached equilibrium. Another theoretical study (Hasegawa, 1969) later elaborated on the mirror theory by including the effects of density and magnetic field gradients and an additional cold particle population in the distribution function. A transverse drift wave mode (drift‐mirror mode) resulted when gradient effects were included, while the cold particle population lowered the condition for instability, making it more favorable. A later theoretical study (Southwood & Kivelson, 1993) showed that kinetic effects of the resonant particle population were non‐negligible, and generated an additional kinetic term in the perturbation of the distribution function. These studies were done in the so‐called quasi hydrodynamic approach, which assumes that wavelengths are much larger than the ion gyroradius. A comprehensive generalization of this approach was developed in Pokhotelov et al. (2002) where solutions of the mirror‐mode dispersion equation were obtained for non‐Maxwellian plasmas. More recent studies (Pokhotelov et al., 2004) have also included the effect of Larmour radius, which creates a wavelength dependence in the growth rate.

Other literature has investigated the relationship between the parallel component of the plasma‐*β* and the plasma anisotropy. Gary et al. (2001) used a theoretically supported relation between the plasma anisotropy and plasma‐*β*_∥_ to empirically derive parameters for the upper bound on the plasma anisotropy due to the electromagnetic proton cyclotron instability in the solar wind. This instability occurs in similar conditions to the mirror/drift‐mirror instability, and was thought to dominate in plasmas with *β* < 1 (Gary et al., 1993; Yoon & Seough, 2012). Later works (Hellinger et al., 2006; Seough et al., 2013) expanded this theoretically calculated, empirically analyzed notion and applied it to the linear theory of proton cyclotron, mirror, parallel firehose, and oblique firehose instabilities, which are all driven by anisotropy in either parallel or perpendicular directions with respect to the background magnetic field. It was found that even at small *β*_∥_, mirror modes can be a controlling mechanism in the plasma. The model was later compared with measurements taken by the wind satellite in the solar wind (Bale et al., 2009), where several magnetic and particle parameters were investigated and compared with the empirically derived values.

Mirror‐mode waves are observed in different space plasmas, including solar wind (Zhang et al., 2008), magnetosheath (Balikhin et al., 2009; Soucek et al., 2008), and inner magnetosphere (Soto‐Chavez et al., 2019). It is worth mentioning that identification of mirror‐mode waves is a difficult problem, and quite often an additional analysis questions the nature of the waves initially identified as mirror waves (see, e.g., Balikhin et al., 2001, 2012; Chisham et al., 1999). A statistical analysis of particle data also indicates that the mirror‐mode instability operates in space plasmas, leading to a relaxation of the mirror‐unstable configurations ([Hellinger et al., 2006] and [Chen et al., 2016]), as defined by linear instability criteria.

In this study, we present a climatology of high‐*γ* plasmas in the inner magnetosphere, where *γ* is the M/D‐M instability condition. We then impose a restriction on plasma‐*β*, similar to the climatology conducted by Cohen et al. (2017), during high‐*γ* events to examine the likelihood of conditions favorable for mirror modes. In Section 2, we describe the spacecraft datasets and define derived data products plasma‐*γ* and plasma‐*β*, as well as explaining the meaning of high‐*γ* and high‐*γ*/high‐*β* events. Section 3 represents a high‐*γ* climatology encompassing the entirety of the Van Allen Probes mission dataset. We show a more restrictive climatological analysis by including the plasma‐*β* parameter in Section 4. The implications of M/D‐M mode formation and transversal through the night‐side inner magnetosphere are discussed in Section 5, followed by concluding remarks in Section 6.

## 2 Instrumentation, Methodology, and Data Analysis

For this study, we have used data from the NASA Van Allen Probes mission, which is composed of twin spacecraft with a comprehensive suite of instrumentation for inner magnetospheric (i.e., *L* < 6) research (Mauk et al., 2013). In situ magnetic field data were obtained from the Electric and Magnetic Field Instrument Suite and Integrated Science (EMFISIS, [Kletzing et al., 2013]) instrument. Particle data were obtained from the Radiation Belt Storm Probe Ion Composition Experiment (RBSPICE, [Mitchell et al., 2013]) instrument. We utilized the 1 s time‐resolved magnetic field data and the 11 s full spin integrated RBSPICE TOF × EH‐Level3PAP particle data with an energy range of 44 keV–597 keV reported for the midpoints of the lowest and highest RBSPICE energy channels, respectively. For this study, we included the pressure contributions from protons exclusively.

We derive two plasma parameters, *β* and *γ*, from the given data. *β* for Sections 3 and 4 will refer specifically to the total *β*. We calculate *β* using the methodology outlined in the Cohen et al. (2017) study, deriving the magnetic field pressure from the EMFISIS dataset and the particle pressures from RBSPICE. For *γ*, we utilize the quasihydrodynamic mirror instability condition in the long wavelength limit (Hasegawa, 1969; Pokhotelov et al., 2004; Soucek et al., 2008),

(1) $$\gamma =\frac{P_{⊥}}{P_{∥}}-\frac{1}{\beta_{⊥}}-1,$$

Here, *P*_⊥_ and *P*_∥_ are perpendicular and parallel (with respect to magnetic field) pressures, so the first term defines plasma temperature anisotropy, and *β*_⊥_ is perpendicular beta. The condition *γ* > 0 corresponds to the plasma having sufficient anisotropy to be unstable with respect to the mirror modes.

For our climatological *γ* analyses, we define a high‐*γ* event as any time the Van Allen Probes had three consecutive TOF × EH and interpolated EMFISIS magnetic field measurements where the derived *γ* > 0 for 33 s. This was done to remove sporadic high‐*γ* measurements which are not representative of the local plasma conditions. In the subsequent analyses, we include measurements where the second M/D‐M instability criterion is satisfied, meaning an event now includes three consecutive measurements where derived *γ* > 0 and derived *β* > 1. The entire event is counted as one if there is any group of three or more consecutive high‐*β* measurements in the event. We have chosen the *β* parameter due to its importance with regards to linear theory. Observations have demonstrated that mirror modes should dominate for *β* > 1, while in *β* < 1 regime, the ion cyclotron instability may dominate even if mirror modes are unstable (*γ* > 0) [see, e.g., (Lacombe & Belmont, 1995; Yoon & Seough, 2012).

## 3 High‐*γ* Climatology of the Inner Magnetosphere

**Figure 1:**
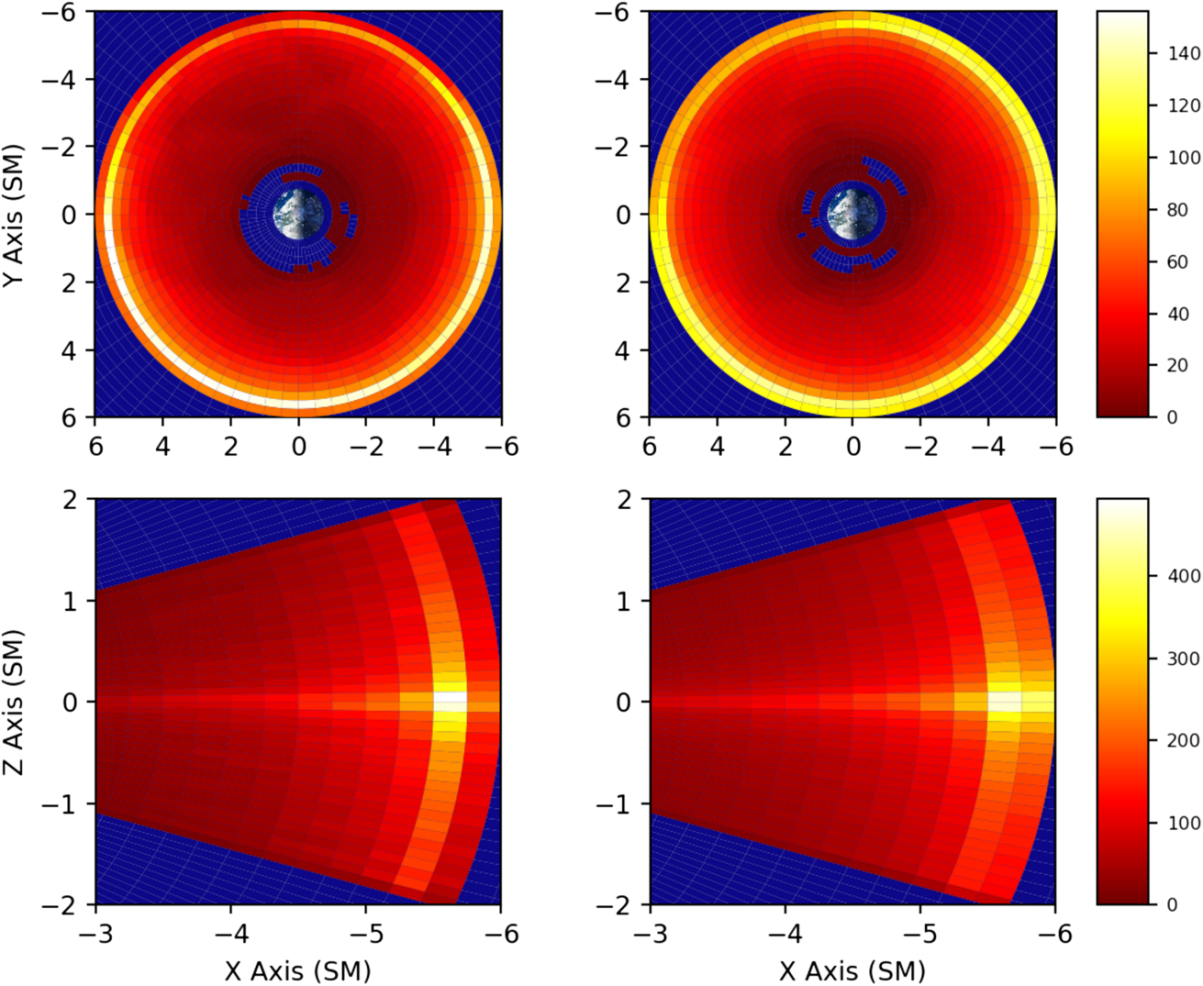
The total number of hours the RBSPICE instrument made measurements for a given spatial location for spacecraft A (left) and spacecraft B (right), in SM coordinates. The two spacecraft are presented separately, due to sampling issues outlined in Section 2. The top row of graphs is a top‐down, *XY*, view on the inner magnetosphere, while the bottom row is a side, *XZ*, view, with the sun to the left. The bottom plots were created by azimuthal integration over all MLT. Areas in blue were not sampled by the mission. RBSPICE, radiation belt storm probe ion composition experiment; MLT, magnetic local time.

We present climatological statistics for the entire lifetime of the Van Allen Probes mission, which is nominally three full MLT orbital precessions, in regards to the M/D‐M instability parameter *γ*. Due to certain asymmetries in the sampling at higher L‐shells, we’ve shown the sampling distribution of the RBSPICE instrumentation in Figure 1, with spacecraft A on the left and spacecraft B on the right. All proceeding climatology plots will be similarly separated. The most likely cause of the asymmetry in coverage was an intermittent high‐voltage discharge issue onboard the RBSPICE instrument on spacecraft A at various times during the course of the mission.

The number of 11 s measurements (i.e., nominal data realizations) on spacecraft A *&* B were 1.325 × 10^7^ and 1.572 × 10^7^, respectively. Although the number of events on spacecraft A will be an underreporting of the true number due to the disproportionate amount of higher L‐shell samples between spacecraft, the climatological analyses still have sufficient statistics to show overall trends. Van Allen Probes reached apogees of *L* ≈ 5.8, with RBSPICE shutoffs imposed at approximately *L* ≈ 3. This shutoff was implemented due to excessive accidentals and instrument charging on the RBSPICE instrument. Near the end of mission, the instrument was left on through perigee for several orbits, accounting for the few stray samples in the lowest L‐shells.

**Figure 2:**
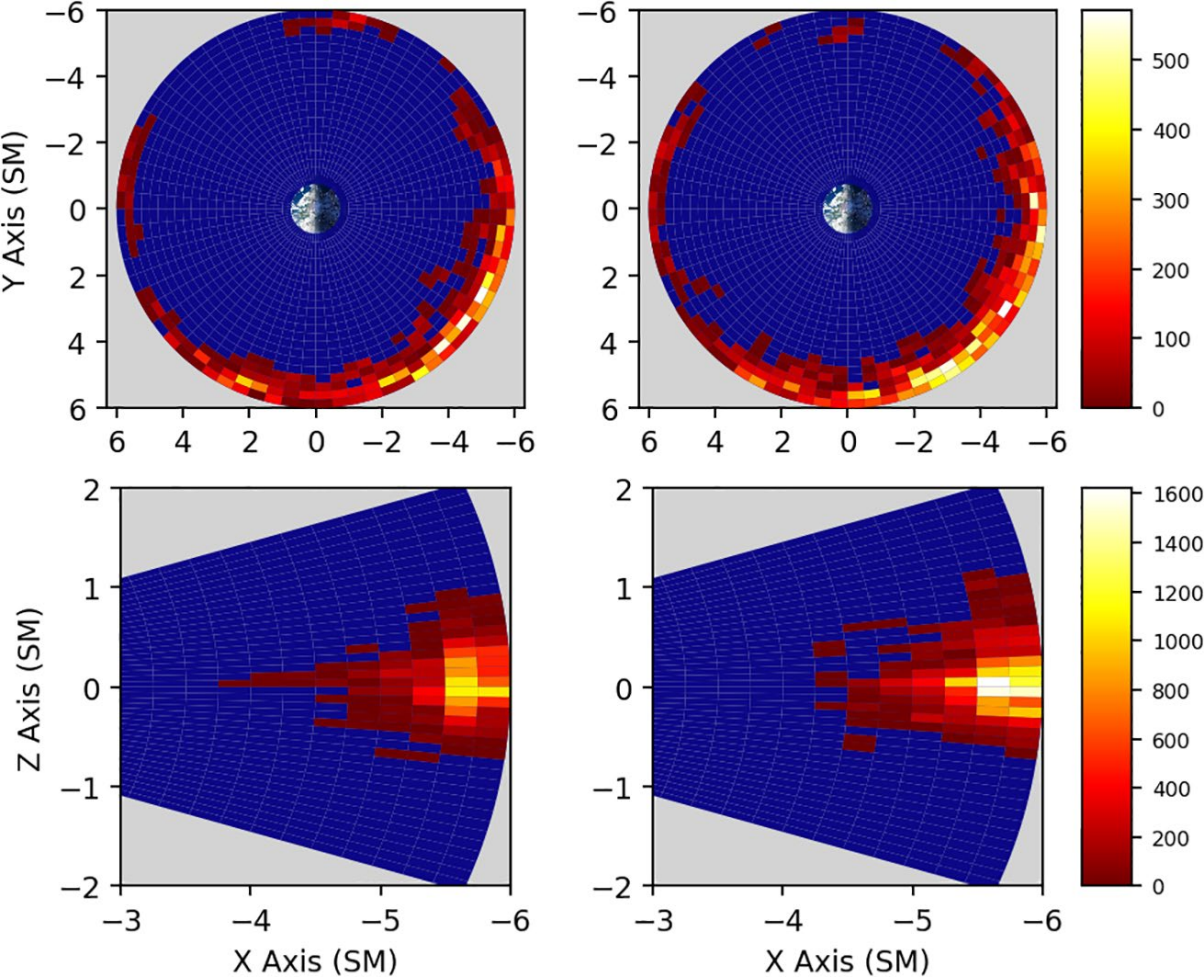
The total number of high‐*γ* occurrences for a given spatial location for spacecraft A (left) and spacecraft B (right) which were part of a set of at least three consecutive measurements where *γ* > 0, indicating the plasma is sufficiently anisotropic for mirror/drift‐mirror waves to form. The bottom plots were created by azimuthal integration over all MLT. MLT, magnetic local time.

Our first statistic is the spatial distribution of high‐*γ* measurements in the X‐Y and X‐Z planes, shown in Figure 2. For the *X*‐*Z* planes of the plot (bottom), each bin has been azimuthally integrated. The disparity between number of observations of high‐*γ* plasma for spacecraft A *&* B, respectively, were 1.6 × 10^5^ and 2.5 × 10^5^. This implies that spacecraft A measured roughly two‐thirds the number of high‐*γ* plasmas, which is in keeping with lack of measurements on A. The distribution of occurrences shows a strong preference for the dusk‐to‐midnight sector, which coincides with the side of the magnetotail that is asymmetrically favored for reconnection and subsequent injection events. It is worth noting that measurements in the dusk‐to‐midnight sector came once approximately every two years. The events we measure are a subset of the events that have actually occurred of the past 7 years in the region. A geosynchronous satellite, for instance, would measure roughly four times the number of events that were measured onboard the Van Allen Probes spacecraft, since these events are localized in the dusk‐to‐midnight sector.

The bottom panes of Figure 2 show strong preference for high‐*γ* plasma formation near the magnetic equator. Figure 1 in its bottom panes illustrates that sufficient measurements were indeed taken across +/− 20° of the magnetic equator. This suggests that a narrow weak field region of the nightside magnetosphere within +/− 8° of the magnetic equator is the likely location for mirror mode formation. Magnetotail injections having equatorially aligned propagation vectors would penetrate deeper into this region. Another noteworthy feature is the slight upward skew of the distribution toward the northern latitudes. It is not clear why this skew appears, and requires further investigation.

**Figure 3:**
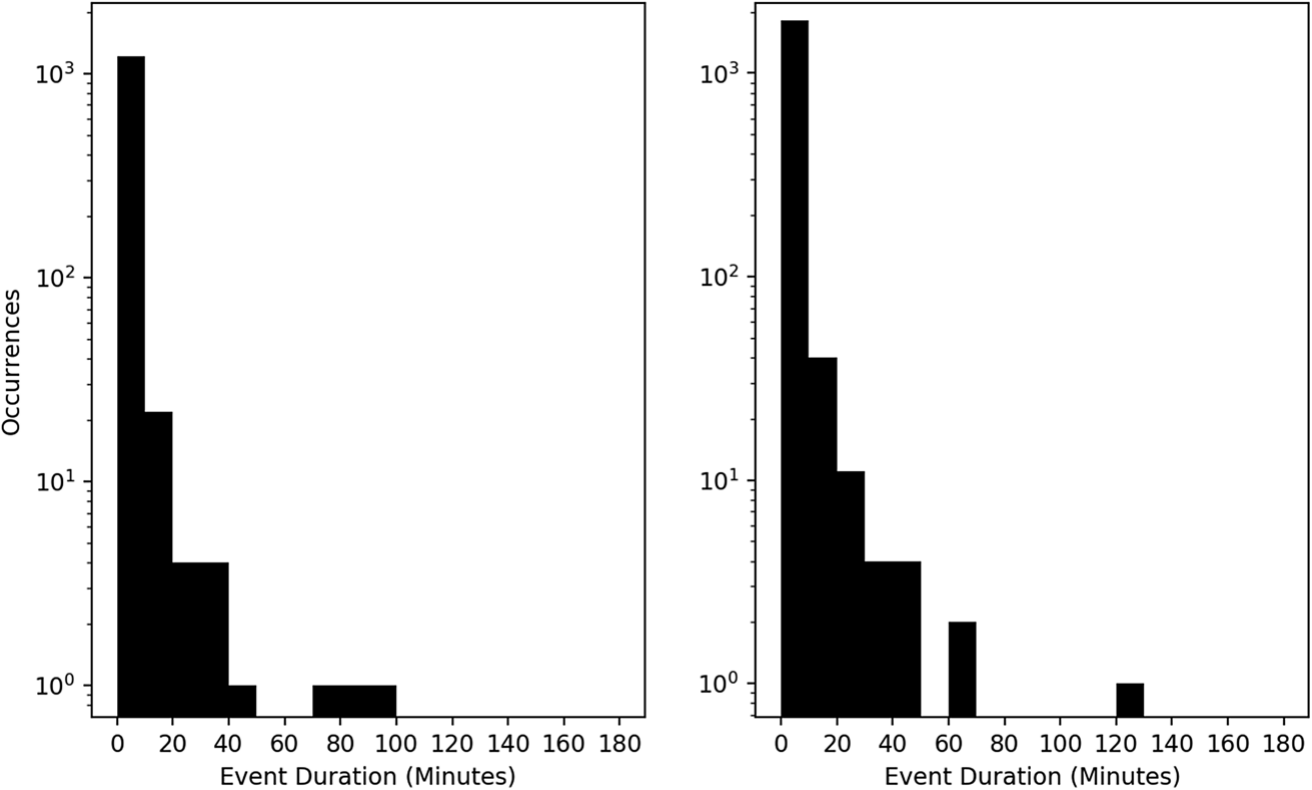
Distribution of occurrences versus duration of high‐*γ* events for spacecraft A (left) and spacecraft B (right). Most events containing at least three consecutive measurements lasted less than 30 minutes, indicating transient features rather than global phenomenon.

The second statistical feature is a histogram of the time duration of individual events, i.e. the duration of any group of three or more consecutive measurements (Figure 3). Any event was at least 33 s in length, with the majority of events occurring within time ranges less than 30 minutes. The majority of these events (96.4*%*/96.6*%* for spacecraft A/B) occurred beyond *L* ≈ 5, where L is taken from the ephemeris data provided with the TOF × EH product from RBSPICE.

**Figure 4:**
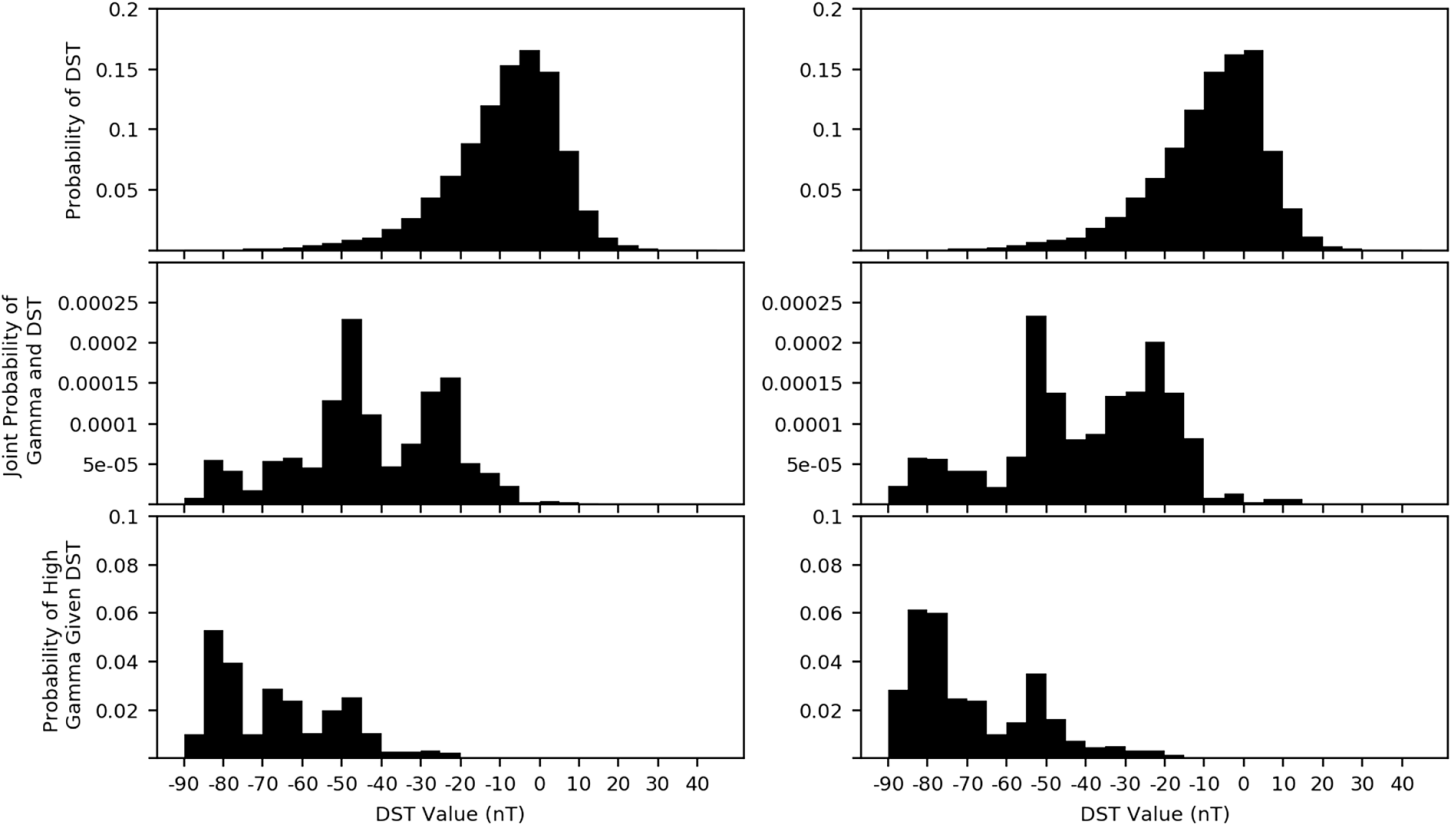
Results of the high‐*γ*/Dst probability analysis. The top panel shows the probability of a given Dst value calculated for the entirety of the mission for spacecraft A (left) and spacecraft B (right). The middle panel is the joint probability of a given Dst value and a high‐*γ* measurement occurring. The bottom panel shows the conditional probability of a high‐*γ* measurement for a given Dst value. *γ* occurrences included in this analysis were part of a high‐*γ* event. Dst.

We suspect that injections from the magnetotail are the main drivers of the observed high‐*γ* regions measured by Van Allen Probes. To give strength to this claim, we initially performed a probability analysis of *γ* measurements compared with the Dst parameter, which is a ground‐based magnetometer derived value representing the amount of plasma in the ring current around Earth's equator. Figure 4 presents a Bayesian analysis, with the top panels revealing that a large portion of Dst measurements fall into quiet time value ranges. The second panels are similar to the *β*/Dst analysis performed in Cohen et al. (2017).

## 4 Joint High‐*β* and High‐*γ* Climatology of the Inner Magnetosphere

We now present analyses of the Van Allen Probes data with regards to the inclusion of the second M/D‐M instability criteria, *β* > 1, into the climatology performed in the previous section. As was discussed, the inclusion of this parameter means in addition to having three consecutive measurements of *γ* > 0, there must also exist three consecutive measurements of *β* > 1 within the event. The *γ* constraint was applied first, followed by *β*. In other words, a single high‐*γ* event having discontinuous sets of three high‐*β* measurements within them is counted as a single event. Had we applied the constraints in the reverse order, our hypothetical event would count as two. The other ordering showed no difference in the climatological analyses, thus this order was chosen.

**Figure 5:**
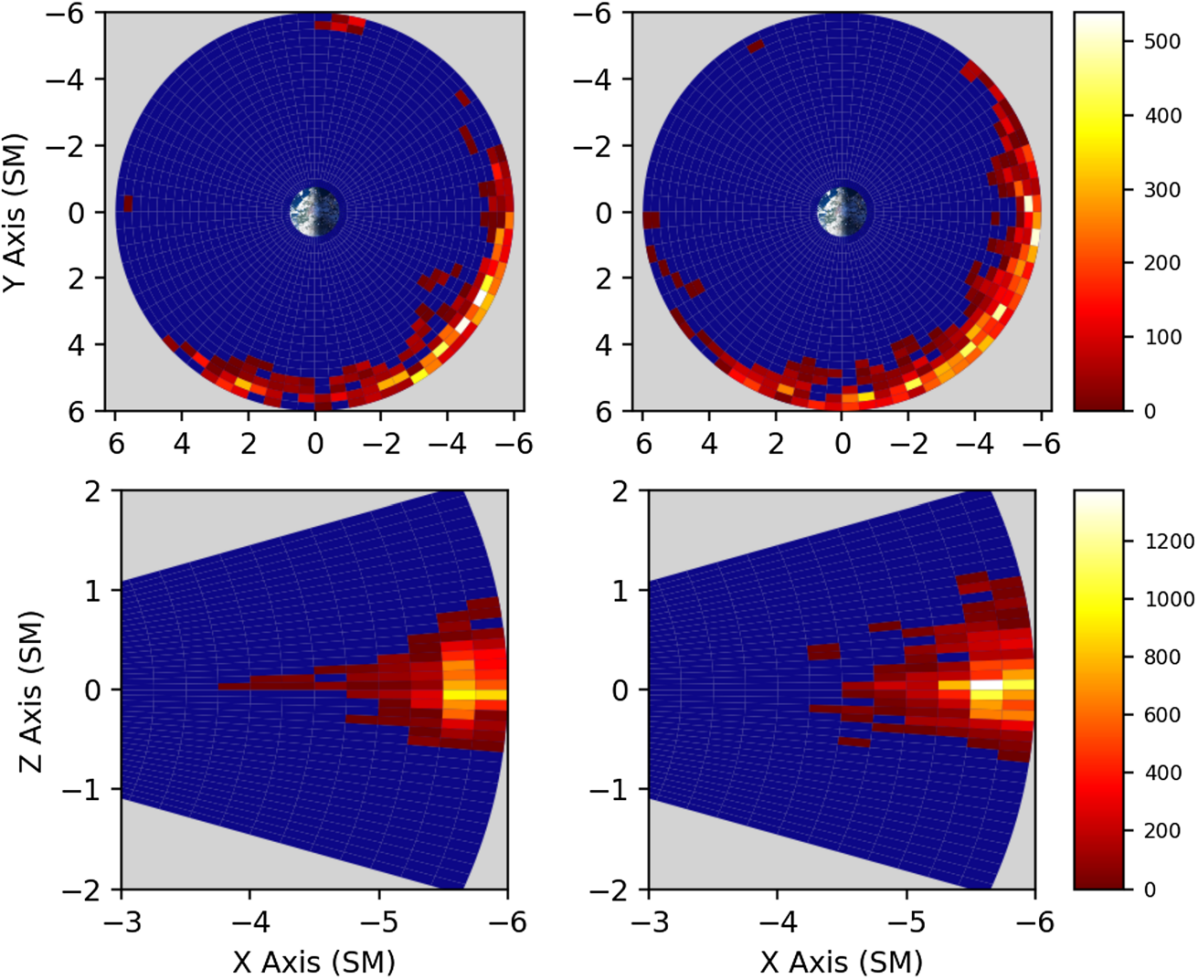
Spatial distribution of occurrences which were included in a high‐*γ*/high‐*β* event, i.e. we first determine if the measurements are part of a high‐*γ* event, then search in that event for three consecutive measurements of high‐*β*. The entire event is included. This was done for spacecraft A (left) and spacecraft B (right). The bottom plots were created by azimuthal integration over all MLT. MLT, magnetic local time.

**Figure 6:**
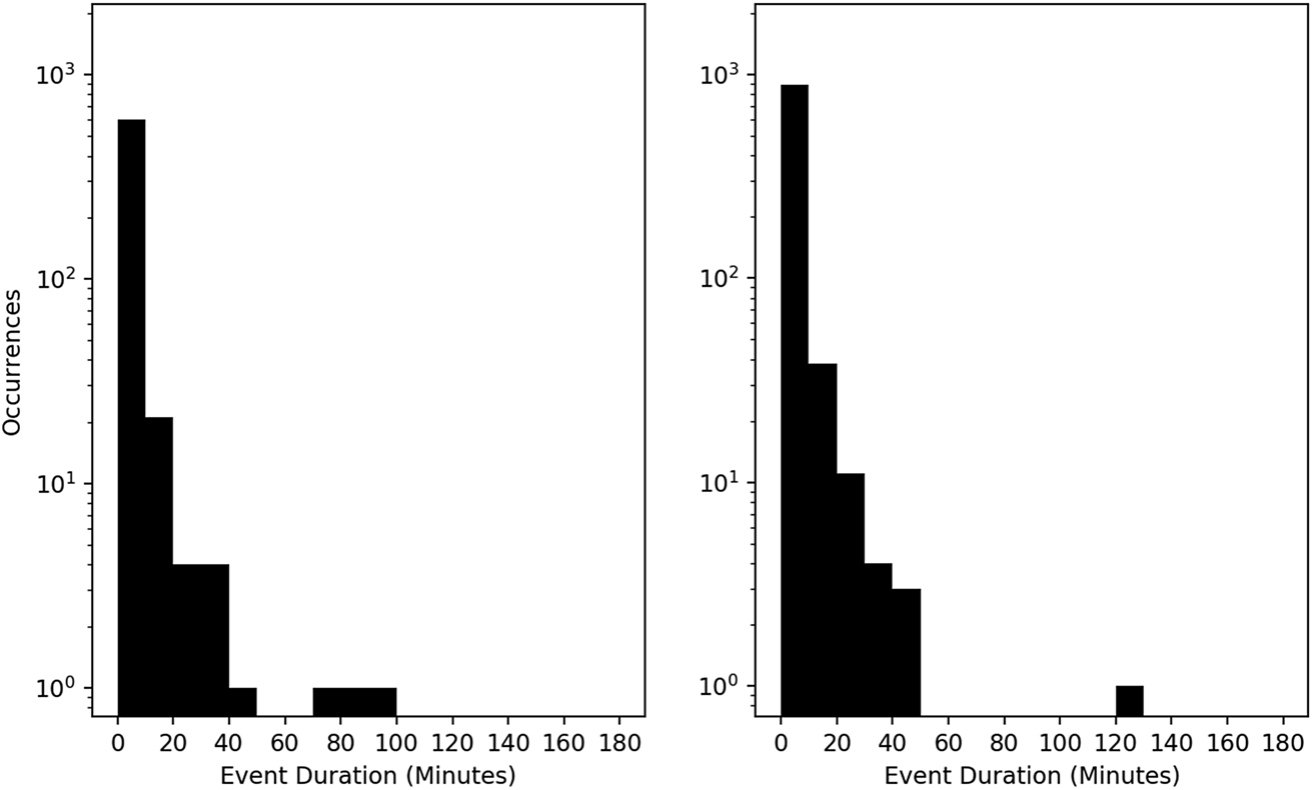
Distribution in duration of high‐*γ*/high‐*β* events for spacecraft A (left) and spacecraft B (right). The inclusion of the *β* restriction creates no significant deviations to the distribution compared to Figure 3.

We follow the previous section's precedent and begin by showing the spatial distribution of high‐*γ* followed by high‐*β* (GfB) plasma measurements in Figure 5. The inclusion of *β* removes the majority of the dayside events, and we recover the spatial distribution presented in Cohen et al. (2017), with the exception of a few pre‐dusk measurements. This indicates that although sufficient anisotropy on the dayside is present, *β* never reaches its threshold value. In the night‐side magnetosphere, magnetic reconnection favors the dusk‐ward side of the magnetotail (Walsh et al., 2014), and is a source of night‐side high‐energy particle fluxes. Cross‐tail electric fields deplete the dusk‐ward ion populations, thinning the current sheet and creating more favorable conditions for magnetic reconnection. Ion diffusion regions (Rogers et al., 2019) and ion injections (Gabrielse et al., 2014) have been observed with similar prevalence in the dusk‐ward magnetotail. Inclusion of *β* in our duration statistics, shown in Figure 6, shows little deviation from the distribution presented in Figure 3. The overall number of measurements has dropped, but since this does not affect the distribution topology, it suggests that both the day‐side and night‐side sources of anisotropy are similar in nature. Further work is needed to determine the validity of this suggestion.

**Figure 7:**
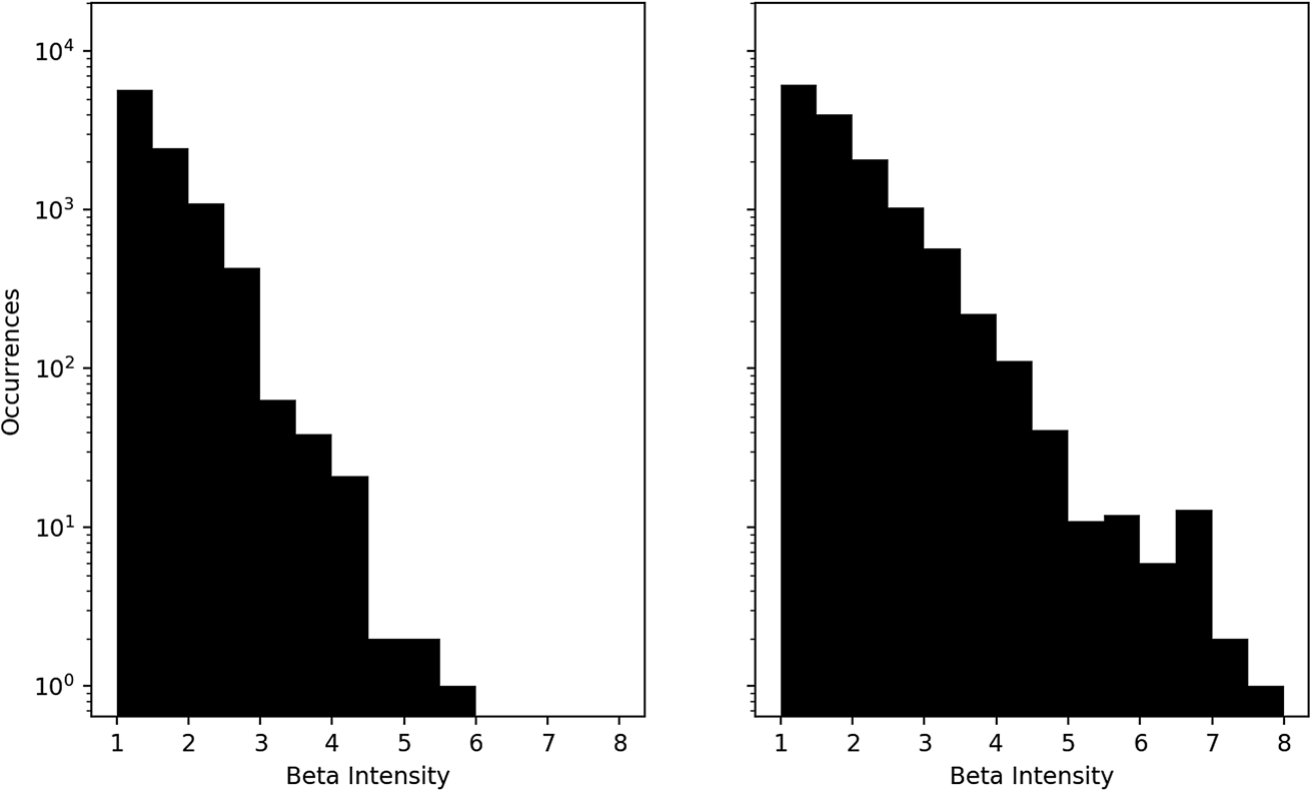
Distribution of occurrences of varying intensities of *β* for spacecraft A (left) and spacecraft B (right) for measurements included in high‐*γ*/high‐*β* events. The majority of *β* values occur near the mirror threshold, but there are instances of *β* reaching as high as eight within the inner magnetosphere.

Figure 7 shows the distribution of *β* values for GfB measurements. In order for a measurement to be counted in one of the histogram bins of Figure 7, its *β* value was between the endpoints of the bin, and the measurement was part of a GfB event. Two aspects of this analysis are noteworthy. First, the majority of the *β* values are close to unity, suggesting high‐*β* events occurring in the dusk‐to‐midnight sector are usually near the frozen‐in/kinetic threshold. Conditions such as these are ideal for the formation of mirror modes. Second, there are cases of *β* values reaching as high as six. These events likely show different turbulence signatures than the normal magnetohydrodynamic‐like plasmas, but their investigation was beyond the scope of this research.

**Figure 8:**
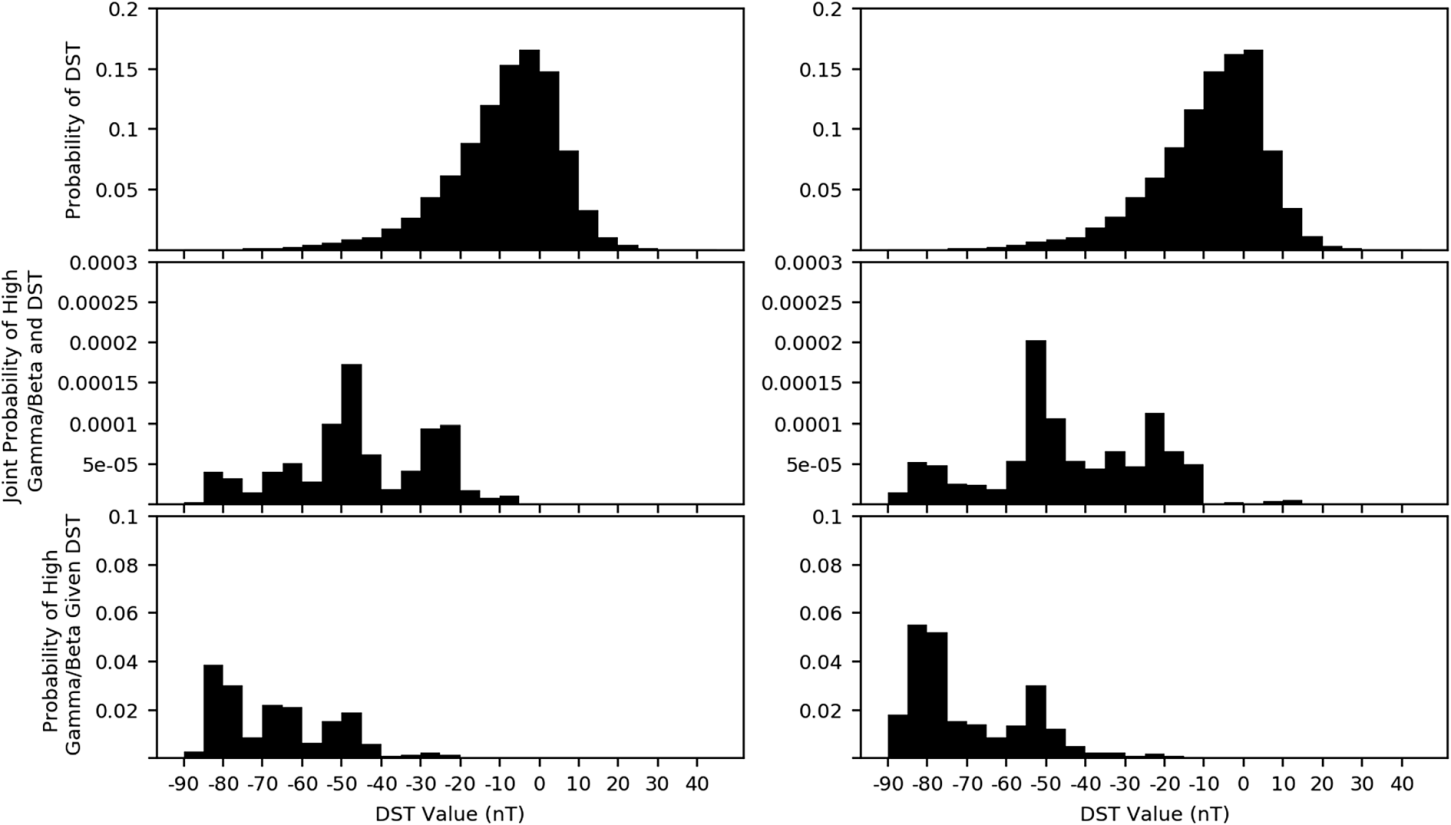
Results of high‐*γ*/high‐*β* measurements compared with Dst. The top panel shows the probability of a given Dst value calculated for the entirety of the mission for spacecraft A (left) and spacecraft B (right). The middle panel is the joint probability of a given Dst value and a high‐*γ*/high‐*β* measurement occurring. The bottom panel shows the conditional probability of a high‐*γ*/high‐*β* measurement for a given Dst value. Dst.

Our final analysis of GfB plasmas, shown in Figure 8, illustrates the correlation between the Dst parameter and GfB plasma measurements. The inclusion of the *β* parameter strongly affects the distribution of Panel 2 of Figure 8 compared to Panel 2 of Figure 4, with most of the measurements made during lower Dst dropping out. The inclusion of the *β* parameter also shows strong semblance to distributions in Cohen et al. (2017). However, Cohen only includes the joint probability. Conclusions drawn Cohen's statistic, which is the joint probability of measuring both a given Dst value and a *β* > 1, showed no strong correlation between the Dst parameter and high‐*β* conditions. We have included a third plot which incorporates the Bayesian conditional probability. There was a skewed sampling of Dst, with the majority of Dst measurements occurring during quiet times. The third panel of Figure 8 reflects this, showing a strong probability of GfB events occurring during times of high Dst values, a result consistent with storm‐time material injection from the magnetotail being the source populations for the GfB occurrences.

## 5 Discussions

We have now built the case for the likely occurrences of M/D‐M modes in the dusk‐to‐midnight inner magnetosphere. We move on to discuss how the formation of mirror structures affect ULF signatures observed by the Van Allen Probes. Our hypothesis is that as material which is observed as either an injection or bursty bulk flow propagates earthward, it eventually reaches a point where the force from the curvature drift of the Earth's dipole field becomes non‐negligible and the plasma begins drifting dusk ward. During this process, small bubbles of plasma whose evolution is subject to the laws of weak kinetic turbulence theory (Treumann & Baumjohann, 2018 and sources therein) begin forming in the otherwise frozen‐in plasma. This formation is commonly referred to as a mirror mode. As they form, they expel magnetic flux from inside the plasma region, creating a boundary in the normal dipole topology. This magnetic field expulsion behaves similarly to classical type II superconductors (Treumann & Baumjohann, 2018). Providing that the mirror mode is able to propagate while remaining a cohesive structure, it will create wakes in the frozen‐in plasma (Treumann et al., 2004) from this expulsion effect, which are candidates for the “stormtime Pc5 ULF wave” disturbances described by Ukhorskiy et al. (2009).

The coherency and propagation direction of such modes is an open scientific question. Another possible secondary source is the coupling of the mirror modes into drift‐mirror waves, which are strongly sinusoidal in nature and may have some relation to the wake modes mentioned previously. Criteria for the distinguishing of drift‐mirror waves from other plasma waves in spacecraft data have only been done on a case study basis (Soto‐Chavez et al., 2019), and further research is required to create a rigorous identification criteria to be used in automated data analyses.

We turn now to an overview of formation and dissipation as mirror modes pass through the inner magnetosphere. Assuming an injection event is the plasma source, mirror bubbles should begin forming once the injection has propagated a relatively short distance. This formation may have some relationship to dipolarization front braking and subsequent lack of penetration into the inner magnetosphere (Dubyagin et al., 2011). The lifetimes of the modes themselves is unknown, however, and the time evolution of a mirror mode once the growth phase ends was a topic of recent PhD dissertation (Ahmadihojatabad, 2016) and is critically dependent on the environmental conditions. This saturation of the mode appears in the dayside magnetosphere as either peaks or dips in 1 s spacecraft magnetometer data (Soucek et al., 2008), but it is uncertain how applicable dayside observations are to the nightside, due to strong differences in the magnetic topology of the regions.

Another unknown feature of this system is how strongly the mirror modes disturb the otherwise frozen‐in plasma in the inner magnetosphere. The propagation of a large structure such as a mirror mode would create disturbances in normal MHD plasma field. Recent studies on conjunction observations between THEMIS and ground‐based Poker Flat Incoherent Scatter Radar (PFISR) (Wang et al., 2020) have shown ULF wave signatures propagating duskward and sunward in a curved path in the ionosphere. Further work is necessary to determine if our effect has any correlation with their results.

Theoretically, such disturbances should be visible in ground magnetometer data as well. However, if this entire region becomes decoupled from the upper and lower hemispheres during very strong storms, field line resonances for the entire dusk‐to‐midnight sector at *L* ≈ 6 might cease to exist at times during the storm evolution. It is also possible that the plasma bubble will act as nonlinear boundary condition, and create novel features in the data.

We have elected not to include the ULF wave data analysis in the present work due to complexities in the Van Allen Probes data, such as a sinusoidal artifact which appears during spacecraft maneuvers which must be characterized and removed. This analysis will be included in a subsequent work. We also stress this study was restrained to the proton *β* and *γ*. There are likely highly dynamic interactions between the hydrogen, helium, and oxygen populations that are present during active times. The inclusion of these species would also require the summation over species to be included in the M/D‐M instability criteria. Also excluded in this study are additional effects derived by Pokhotelov et al. (2004).

## 6 Conclusions

We have presented an updated climatology, which supports the observations and conclusions made by Cohen et al. (2017), with two important addendums. The dependence (or lack thereof) of β on the Dst index which was previously reported changes when considering which Dst values were sampled. Also, due to the spacecraft having flown through more orbits, the spread in the region where high‐*β* plasmas occur has increased. We found sufficient anisotropies over half of all MLT sectors for mirror mode formation, but high‐*β* conditions are only present in the dusk‐to‐midnight sectors. We observed that in the dusk‐to‐midnight sector, most events which had necessarily high anisotropy also had high *β*, indicating the plasma to be preferentially M/D‐M mode unstable.

## Data Availability

RBSPICE data are available at http://rbspice.ftecs.com/Data.html. EMFISIS data are available at http://emfisis.physics.uiowa.edu/data/index.
